# O002. Did Picasso and De Chirico really suffer from migraine auras?

**DOI:** 10.1186/1129-2377-16-S1-A192

**Published:** 2015-09-28

**Authors:** Carlo Lisotto, Federico Mainardi, Ferdinando Maggioni, Giorgio Zanchin

**Affiliations:** Headache Centre, Department of Neurosciences, University of Padua, Padua, Italy; Headache Centre, Hospital of Venice, Venice, Italy

## Background

Some authors have suggested that migraine aura may have represented an inspiration for unusual and recurrent features of paintings by Pablo Picasso and Giorgio De Chirico. A migraine hypothesis was formulated for Picasso's art, based on aura-like patterns, such as illusory vertical splitting and shifts of the eyes, even if the authors explicitly stated that they did not find any supportive information in Picasso's biographies [1]. As for De Chirico, the jagged effect of water, the spiky silhouette of a knight, a black sun motif intruding into an interior scene were interpreted to be evoked by aura. Other descriptions included sparkling, dazzling, dancing or flickering lights, fire rings, stars, and dancing lines [2]. We looked for further evidence to examine in depth this hypothesis.

## Materials and methods

We read Picasso's biographies and De Chirico's autobiography, evaluating their extremely numerous pictorial works, in their different life artistic phases.

## Results

In Picasso's biographies there is no report of the artist suffering throughout his long life from any particular illness, migraine included. Thus, the phenomenal similarity of his works with certain hallucinatory form constants, such as mosaic vision, illusory vertical splitting or metamorphopsia, cannot permit to infer the presence of inspiring visual experiences of a specific aetiology such as migraine, if such diagnosis is not supported by additional evidence. The style characterizing the portraits of female models that could resemble migraine aura seems to be just an evolution from the previous Cubist period. As for De Chirico, he started painting the black sun motifs when he was over 80, an age when the occurrence of aura is extremely unusual. Conversely, other authors objected that the available evidence gathered from the artist's autobiography suggests a diagnosis of epilepsy. De Chirico did not describe typical migraine headaches and several of his ictal symptoms are rare in migraine, but frequently encountered in temporal lobe epilepsy [3].

## Conclusions

To prevent an inflated use of the said diagnostic attribution in pathographic studies of celebrated artists, a methodological standard should be fulfilled in studies of migraine aura as artistic inspiration. We do not wish to subscribe to neurological reductionism, but we believe that art criticism should ideally suggest neurobiological links between the cluster symptoms of a given artist and the key characteristic of the artist's style. The diagnosis of migraine should be supported by semi-/autobiographical writings and/or the observations of contemporaries in addition to the evidence derived from the analysis of the artworks.
